# Supplementary material for: Medicinal Plants and Their Traditional Uses in Local Communities around Cherangani Hills, Western Kenya
Source: Plants (Basel). 2020 Mar 5;9(3):331. doi: 10.3390/plants9030331 (PMC7154930; doi:10.3390/plants9030331)
Supplement: Supplementary file 1 [file plants-09-00331-s001.pdf]

Supplementary material 1

Table S1. Medicinal plants of Cherangani hills, their habit, habitat, part used, method of preparation and administration, and references. Abbreviations for voucher specimens: FOKP- Flora of Kenya Project; SAJIT- Sino Africa Joint Investigation Team; YMM = Yuvenalis Morara Mbuni; IR = Interview results.

Family, Botanical name	Local name Marakwet = (M) ; Pokot = (P); Luhya = (L)	Voucher no.	Habit	Habitat	PU	Method	General plant uses	References
Acanthaceae, *Acanthus eminens* C.B.Clarke	Tegilde (M), Liragalu (L)	FOKP 1002	S	Bushland	L, W	Burning	Whole plant burnt and ash is used for the treatment of spleen diseases and joint pains.	15, 37, 39, 41, 50, 67
Acanthaceae, *Asystasia mysorensis* (Roth) T.Anderson	Oringogwo (P), Kisuvu (L)	SAJIT 005148	H	Escarpment Valley	L	Crushing	Crushed leaves infusion drunk for colds, headache, and malaria.	IR, 15,
Acanthaceae, *Blepharis maderaspatensis* (L.) B.Heyne ex Roth	Chemo (P)	SAJIT-Z0044	H	Bushland, Grassland	L	Pounding	Pounded leaves and then infusion used for eye infections.	15, 89
Acanthaceae, *Justicia flava* (Forssk.) Vahl	Chebarus (M), Uhululu (L)	SAJIT 004725	H	Escarpment, Bushland	R	Boiling, Chewing, Burning	Root decoction drunk for abdominal pains and diarrhea. Roots chewed for coughs. Leaves ash used for emetic and oedema.	10, 15, 41, 46

Acanthaceae, *Thunbergia alata* Bojer ex Sims	Chelolony (M), Ketenai (P), Tsindelesia (L)	FOKP 11275	H	Bushland	L, W	Crushing	Crushed leaf juice taken orally for mouth and tongue infections. Whole plant decoction drunk for backache and joint pains.	15, 19, 39, 41, 46 70
Amaranthaceae, *Achyranthes aspera* L.	Chesrimion (M), Chesrimion (P), Lusayi (L)	FOKP 11345	H	Escarpment	R, L	Boiling, Pounding	Root decoction drunk for constipation. Pounded roots used to cure gonorrhea. Pounded leaves added to warm water, bathe for wounds and skin diseases.	15, 25, 32, 39, 51, 70, 86, 91
Amaranthaceae *Aerva lanata* (L.) Juss.	Chepskut (M)	FOKP 11485	S	Woodland	R, W	Burning, Boiling	Licking ash from the burnt whole plant to treat ulcers and stomachache. Root infusion for Malaria.	15, 70, 71
Amaranthaceae, *Chenopodium opulifolium* Schrad. ex W.D.J.Koch & Ziz		FOKP 11342	H	Cultivated	L	Boiling	Leaf decoction drunk for liver diseases and diarrhea.	50, 91
Amaranthaceae, *Cyathula cylindrica* Moq.		SAJIT 007115	H	Bushland	B, R	Boiling	A decoction of the bark of the root drunk as a remedy for malaria and leprosy.	15, 41
Amaranthaceae, *Cyathula uncinulata* (Schrad.) Schinz	Kimnamgwe (M), Lusai (L)	FOKP 1126	C	Escarpment Bushland	R, L, Fr	Boiling	Boiled root decoction used to induce pregnancy. Leaf decoction for skin rashes. Fruits eaten for abdominal pains.	IR, 15, 19, 39

Anacardiaceae, *Lannea fulva* (Engl.) Engl.	Lolotwa (M), Lolotwo (L), Lumubumbu (L)	FOKP 11335	S	Escarpment Woodland, Grassland	B, R	Boiling	Roots decoction for chest pains and coughs; bark infusion is employed against stomachache.	IR, 10, 15, 19, 71
Anacardiaceae, *Lannea triphylla* (Hochst. ex A. Rich.) Engl.	Korut (M), Moino (P)	YMM/19/133	T	Escarpment	R, B	Boiling, Chewing	Bark and root concoction used against liver diseases, dysentery, rheumatism, body swelling, and malaria. Fresh bark chewed for colds.	IR, 15, 71
Anacardiaceae, *Ozoroa insignis* Delile	Mutung'wa (M), Kromwo (P)	SAJIT 004740	T	Escarpment Woodland, Grassland	R, Br, B	Boiling, Crushing	Root, branches, and bark concoction administered against diarrhea, stomachache, kidney, and diseases. Bark crushed and extract applied on snake bite.	IR, 54, 71, 86, 92
Anacardiaceae, *Rhus longipes* Engl.	Seriat (M),	FOKP 11308	T	Escarpment, Woodland, Grassland	R	Boiling	Decoction of boiled roots used in the treatment of stomachache and headache. Root infusion used for the treatment of influenza.	IR, 19, 94
Anacardiaceae, *Rhus natalensis* Bernh. ex C.Krauss	Seria (M), Siryewo (P), Busangula (L).	SAJIT 007047	S	Thickets, Woodland, Grassland	R, L	Boiling, Pounding	Root decoction drunk for diarrhea, abdominal pains and gonorrhea. Leaves used as remedy to tonsillitis, boils, joints, and coughs.	IR, 15, 19, 39, 41, 46, 62, 68
Anacardiaceae, *Rhus tenuinervis* Engl.	Seriat (M),	FOKP 11740	S	Wooded grassland, Bushland	L	Chewing	Leaf juice from leaves eradicates heartburn.	15, 92

Anacardiaceae, *Rhus vulgaris* Meikle	Seriat (M), Siriowa (P)S ungula (L)	FOKP 11650	T	Thickets, Wooded grassland,	R, Br, L,	Boiling	Branches and leaves decoction used for cleaning wounds. Roots decoction used for gonorrhea and assists women to deliver easily.	47, 50, 67, 68
Anacardiaceae, *Sclerocarya birrea* (A.Rich.) Hochst.	Arolwa (M), Roluwo (P),	YMM/19/099	T	Highland, Escarpment	R, B	Boiling, Pounding	Bark chewing used to treat toothache. Root decoction is taken orally for stomachache, constipation, dysentery, malaria, diarrhea, fever, ulcers, enlarged spleen, and liver. Leaves used for heartburn. Fruits are eaten for colds.	IR, 15,
Annonaceae, *Uvaria leptocladon* Oliv.	Mulkushu (M), Mulkusion (P)	FOKP 11447	S	Bushland	R	Boiling	Root decoction drunk for Gonorrhea and as a remedy for dysentery.	71
Annonaceae, *Uvaria scheffleri* Diels	Murguiyo (M), Tamrenwo (P)	FOKP 11469	T	Escarpment, Woodland	R, B	Boiling	Decoction of root and bark used against Malaria.	IR, 15, 19, 51, 71
Apiaceae, *Heteromorpha trifoliata* (H.L.Wendl.) Eckl. & Zeyh.		FOKP 11567	S	Escarpment Grassland	R	Boiling	Root decoction drunk to treat syphilis.	IR, 39
Apiaceae, *Peucedanum aculeolatum* Engl	Borio (M)	FOKP 11398	H	Highland	R	Boiling	Root decoction to stimulate the evacuation of the bowels.	74

Apocynaceae, *Acokanthera oppositifolia* (Lam.) Codd	Kelyo (M)	FOKP 1097	S	Woodland	L, R	Pounding	Infusion of pounded roots and leaves used for headache	15, 91
Apocynaceae, *Acokanthera schimperi* (A.DC.) Schweinf.	Kelwon (M), Kalyan (P)	FOKP 11306	T	Woodland Grassland	R, B	Boiling	Infusion of root and bark used to treat syphilis.	10, 15, 41, 43
Apocynaceae, *Carissa edulis* (Forssk.) Vahl	Leketetwo (M), Logetetwa (P)	YMM/19/059	S	Escarpment Bushland	R, B	Boiling	Concoction of roots and bark used as pain killer, against dysentery, fever, malaria, and as an aphrodisiac.	IR, 41, 42, 43, 54, 70, 92
Apocynaceae, *Carissa spinarum* L.	Loketetwo (P) Eshikata (L)	FOKP 1109	S	Highland	R, L	Boiling	Root decoction used for malaria, chest pain, arthritis, gonorrhea, epilepsy, and abdominal pains. Leaf decoction used for breast cancer.	IR, 15, 50, 54, 67
Apocynaceae, Cynanchum defoliascens K.Schum	Chepanyinda (P)	YMM/19/102	C	Escarpment, Bushland	R	Boiling	Boiled root drunk to strengthen children.	IR, 70
Apocynaceae*,* *Gomphocarpus fruticosus* subsp. *flavidus* (N.E.Br.) Goyder	Chepkors (P)	YMM/19/093	S	Highland Grassland	L	Boiling	Leaf infusion used against fevers and abdominal pains.	IR, 15, 19, 71, 91

Apocynaceae, *Landolphia buchananii* (Hallier f.) Stapf		FOKP 1270	C	Riverine	Fr	Chewing	Eating ripe and unripe fruits clears mouth and tongue infections.	34, 38, 39
Apocynaceae *Mondia whitei* (Hook.f.) Skeels	Mukombelo (L)	FOKP 1718	C	Bushland, Riverine	R	Chewing	Chewing roots as an aphrodisiac, appetizer, and fertility enhancer. Treatment of indigestion, heartburn, kidney ailment, asthma, and gonorrhea.	IR, 15, 22, 61
Apocynaceae, *Periploca linearifolia* Quart.Dill. & A.Rich.	Sinindet (M), Sinendet (P),	SAJIT 005059	C	Highland	Br, L	Rubbing	Branches and leaves latex used to treat gonorrhea, and syphilis.	IR, 11, 15, 18, 36, 38, 40
Apocynaceae, *Tabernaemontana stapfiana* Britten	Kibararia (M), Kunandere (L)	SAJIT 006887	T	Highland	B, R	Boiling	Bark and root decoction used to relieve headache, stomachache, and breast cancer.	IR, 34, 38, 40, 44
Aquifoliaceae, *Ilex mitis* (L.) Radlk.	Seger (M)	SAJIT 006912	T	Escarpment	B	Chewing	Chewing bark enhances fertility in women and bark decoction drunk for abdominal pains.	34
Asparagaceae, *Asparagus falcatus* L.		FOKP 11699	C	Highland Woodland	R	Boiling	Decoction of roots drunk for gonorrhea and stomachache ailments.	15, 68

Asparagaceae, *Asparagus flagellaris* (Kunth) Baker	Kapngai (M)	YMM/19/106	S	Escarpment, Bushland	R	Boiling, pounding	Decoction of roots drunk for stomachache and syphilis. Fruits are eaten for conjunctivitis.	IR, 15, 68
Asparagaceae, *Asparagus racemosus* Willd.	Kabungai (M)	FOKP 11699	S	Escarpment, Bushland	R	Boiling	Root decoction drunk for indigestion, kidney diseases, and gonorrhea.	IR, 18, 36, 39, 40, 68
Asparagaceae, *Chlorophytum subpetiolatum* (Baker) Kativu	Matib sorkor (M)	YMM/19/130	H	Grassland, Woodland	R	Boiling	Root decoction drunk to reduce madness.	IR
Asparagaceae, *Dracaena afromontana* Mildbr.		FOKP 1070	T	Highland	R	Boiling	Root decoction used for chest pains.	IR
Aspleniaceae, *Asplenium loxoscaphoides* Baker		SAJIT 007117	H	Montane	L	Crushing	Infusion of crushed leaves used for the treatment of sore throat.	IR
Asteraceae, *Acanthospermum glabratum* (DC.) Wild	Kwilisungura (L)	SAJIT Z0007	H	Cultivated	L	Pounding	Pounded leaves powder applied on boils.	34
Asteraceae, *Acmella caulirhiza* Delile		FOKP 1230	H	Valley	R, L, Fl	Chewing, Pounding, Crushing	Chewing roots heals sore throat. Pounded leaves sap assists thrush in children. Crushed whole plant juice is drunk for stomachache and	15, 32, 36, 47, 68, 72

							diarrhea. Flower head infusion used as ear-drop.	
Asteraceae, *Ageratum conyzoides* (L.) L.	Ilusa (L)	FOKP 11495	H	Cultivated	R, L	Boiling, pounding	Root and leaves concoction drunk for chest pains, stomachache, and coughs. Leaf infusion treats sore eyes.	15, 38, 46, 47, 51, 61, 73
Asteraceae, *Artemisia afra* Jacq. ex Willd.	Sesimwa (M)	SAJIT 005114	H	Highland, Escarpment Grassland	L, W, R	Burning, Crushing, Boiling	Root decoction drunk for intestinal worms. Leaves decoction drunk for colds, influenza, malaria, and fever. Crushed leaves juice used as an ear drop. Ash from burnt whole plant used for treating abdominal pain.	IR, 15, 18, 36, 46
Asteraceae, *Aspilia pluriseta* Schweinf. ex Schweinf.	Shilambila (L)	FOKP 11492	S	Bushland, Grassland	L	Pounding	Pounded leaves juice applied on skin and cut wounds.	15, 43, 46, 47, 51, 71, 90
Asteraceae, *Berkheya spekeana* Oliv.	Kibitet (M), Shirakala (L)	SAJIT 005124	H	Woodland, Grassland	W	Burning	Ash from burnt whole plant is licked for indigestion.	IR, 15, 18
Asteraceae, *Bidens pilosa* L.	Chepkotiwot (M), Chepaswoi (P), Ologoye (Luhya)	FOKP 11434	H	Highland, Woodland	R, L	Boiling, Squeezing, Pounding, Chewing, Crushing	Leaf Crushed, then tied to the fresh cuts to stop bleeding, Root infusion drunk for coughs. Pounded leaves juice assists children with constipation problems, stomachache, and eradication	15, 44, 51, 73, 71, 86, 89, 90, 91

							of intestinal worms. Root and leaf concoction drunk for skin cancer.	
Asteraceae, *Blumea axillaris* (Lam.) DC.	Cheptakar (P)	FOKP 11366	H	Escarpment	R	Boiling	Root decoction drunk for gonorrhea.	IR, 15,
Asteraceae, *Conyza bonariensis* (L.) Cronquist	Chepkoi (P)	FOKP 11338	H	Cultivated	R, L	Boiling	Leaves infusion used to cure fever. Root decoction drunk for liver diseases and stomachache.	IR, 15, 36
Asteraceae, *Conyza newii* Oliv. & Hiern	Kipkosum (M)	SAJIT 007098	S	Escarpment	R, L	Chewing	Leaves and roots chewed for chest pains.	15, 18
Asteraceae, *Conyza pyrrhopappa* Sch.Bip. ex A.Rich.	Kiroria (M), Kanuni (P)	SAJIT 006854	S	Upland, Bushland	R, L	Pounding, Boiling	Roots ground and applied in the throat for sore throat and heals tonsillitis. Pounded leaves infusions drunk for indigestion and malaria. Boiled roots are used for stomach problems and influenza.	15, 18, 36, 47
Asteraceae, *Crassocephalum picridifolium* (DC.) S.Moore	Bunimkubwa (L)	SAJIT 004806	H	Riverine	L, R	Crushing, Boiling	Crush leaves, add water and bathe to enhance strength in pregnancy. Root decoction drunk for stomachache.	15, 47, 91

Asteraceae, *Crassocephalum vitellinum* (Benth.) S.Moore	Terkekwo (M)	FOKP 981	H	Woodland	Fl, L	Rubbing, Boiling	Rubbing flower to the eye removes tiny particles. Leaves decoction drunk by pregnant women to give them strength during child birth.	15, 86
Asteraceae, *Helichrysum forskahlii* (J.F.Gmel.) Hilliard & B.L.Burtt		SAJIT 006805	H	Grassland	R	Pounding	Pounded roots infusion drunk for nausea and vomiting.	IR
Asteraceae, *Helichrysum globosum* Sch.Bip.	Keremut (P)	FOKP 11478	H	Grassland	R	Boiling	Root infusion drunk when placenta delays in coming out after child birth.	IR, 15,
Asteraceae, *Helichrysum odoratissimum* (L.) Sweet	Inamalaba (L)	FOKP 11537	S	Highland	R, L, Br	Pounding,	Crushed roots cure coughs. Pounded leaves and branches applied on wounds.	15, 44, 91
Asteraceae, *Helichrysum schimperi* (Sch.Bip. ex A.Rich.) Moeser		FOKP 11340	S	Highland	L	Chewing	Leaves are chewed as a cough remedy.	IR, 15,
Asteraceae, *Lactuca inermis* Forssk.		FOKP 11348	H	Highland	R	Boiling	Root decoction used as remedy for gonorrhea and syphilis.	IR, 15, 44

Asteraceae, *Laggera brevipes* Oliv. & Hiern	Litumusi (L)	SAJIT 006881	S	Grassland,	L	Boiling	Leaf decoction drunk for treatment and prevention of measles.	IR, 15,
Asteraceae, Laggera crispata (Vahl) Hepper & J.R.I.Wood		SAJIT 006801	S	Grassland	R	Chewing	Root chewing used for hiccups and colds.	IR, 15, 67
Asteraceae, *Melanthera scandens* (Schumach. & Thonn.) Roberty	Kisangwa (M), Kwa mino(L)	FOKP 11755	S	Highland	W, L	Burning, Boiling	Whole plant decoction drunk for indigestion; leaf ash used as a mouth wash for infected teeth.	15, 18, 44, 91
Asteraceae, *Microglossa densiflora* Hook.f.		SAJIT 006797	S	Bushland	L	Boiling	Leaf decoction drunk for stomachache in children.	IR
Asteraceae, *Psiadia punctulata* (DC.) Vatke	Konocho (M), Shiro (L)	FOKP 11465	S	Escarpment, Grassland, Bushland	R, L, Fl	Boiling, pounding	Root decoction drunk for malaria, coughs, sore throat, sterility, and as an aphrodisiac. Powdered leaves are used for colds. Flower decoction heals afterbirth pains.	IR, 15, 71
Asteraceae, *Tarchonanthus camphoratus* L.	Lelekwo (M)	YMM/19/073	T	Highland, Escarpment	L, Br	Boiling	Concoction of leaves and branches drunk for asthma, bronchitis, fever, and gastrointestinal diseases.	IR, 15

Asteraceae, *Vernonia adoensis* Sch.Bip. ex Walp.	Kiptamit (M), Lusutsa (L)	FOKP 11386	S	Highland, Escarpment	R, L	Boiling	Leaves infusion drunk for malaria. Root decoction drunk for TB and gastrointestinal disorders, heart and kidney diseases, also assists in children's stomachache.	IR, 15, 46
Asteraceae, *Solanecio mannii* (Hook.f.) C.Jeffrey	Yergekwa (M),	Mbuni 193	T	Cultivated	R, L	Pounding, Boiling	Pounded leaves rubbed on snake bite wounds. Roots decoction drunk for dysentery and indigestion.	15, 36, 38, 47
Asteraceae, *Tarchonanthus camphoratus* L.	Akwegeyan (P),	FOKP 11297	S	Bushland	L	Boiling	Concoction of leaves and twigs drunk for asthma, bronchitis, fever, and gastrointestinal disorders.	15, 90
Asteraceae, *Tridax procumbens* (L.) L.		SAJIT Z0038	H	Grassland	L	Chewing	Leaves chewed as a remedy for malaria and stomachache.	IR, 15,
Asteraceae, *Vernonia amygdalina* Delile	Ketpomukang (P), Lisulushita (L)	FOKP 10934	S	Cultivated	R, L	Pounding	Pound leaves and roots juice drunk as a remedy for fever and malaria.	15, 81, 61, 86
Asteraceae, *Vernonia holstii* O.Hoffm.		FOKP 11454	S	Woodland	R	Boiling	Root decoction drunk to relieve abdominal pains.	IR, 15,
Asteraceae, *Vernonia hymenolepis* A.Rich.	Kiptamit (M)	FOKP 11402	S	Bushland	R	Boiling,	Root decoction used for the treatment of abdominal pains.	IR, 15, 18, 47

Asteraceae, *Vernonia lasiopus* O.Hoffm.	Lisulushita (L), Muhasha (K)	YMM/19/006	H	Cultivated	R, L	Boiling	Root and leaf concoction drunk for malaria, stomachache, and sore throat.	IR, 15, 34, 44, 73, 86
Balsaminaceae, *Impatiens sodenii* Engl. & Warb.	Gororot (M)	SAJIT 004733	H	Escarpment	R	Boiling	Root decoction used as a purgative.	15, 18, 70
Balsaminaceae, *Imptiens tinctoria* A.Rich	Kibolio (M)	SAJIT 004789	H	Highland	R	Boiling, chewing	Root decoction used for the treatment of abdominal pains. Chewing stems eliminates mouth infections, and treats sore throat.	15, 18, 34, 36
Basellaceae, *Basella alba* L.	Rachan (P), Inderema (L), Kiraita (M)	FOKP 982	C	Riverine	L	Boiling	Leaf decoction increases lactation in mothers, cure abdominal pains, regulates monthly periods in women and relieves chest pains.	15, 34, 36, 39, 40, 61, 73, 71
Berberidaceae, *Berberis holstii* Engl.	Kapchebin (M)	SAJIT 007059	S	Escarpment, Bushland	R, B	Boiling, Pounding	Bark of root peeled, pounded and then applied to wounds. Root decoction for curing stomachache.	IR, 15
Boraginaceae, *Cordia africana* Lam.	Bonbonwa (M)	SAJIT 005062	T	Riverine, Woodland Grassland	B	Boiling	Bark decoction drunk for gonorrhea and syphilis.	15, 34, 36, 47
Boraginaceae, *Cordia monoica* Roxb.	Msasa (K)	SAJIT Z0060	S	Bushland	R, L	Boiling	Root decoction drunk for malaria and to control vomiting in children. Leaves juice are used for eye ache.	IR, 15,

							Leaf extract removes the retained placenta.	
Boraginaceae, *Ehretia cymosa* Thonn.	Morori (M), Ponponat (P), Shekhutu (L)	SAJIT 007051	S	Highland	B, R, L,	Boiling	Root and leaves decoction used as an aphrodisiac and stomachache and ulcers. Leaf juice extract applied on wounds.	15, 34, 38, 40, 39, 42, 46, 67, 73
Boraginaceae, *Trichodesma zeylanicum* (Burm.f.) R.Br.		FOKP 11281	H	Bushland	R, L	Boiling, Chewing	Leaves powder used for snake bite. Root decoction used for TB, ulcers and stomachache.	IR, 15,
Burseraceae, *Commiphora eminii* Engl.		SAJIT Z0062	T	Highland	R, B	Boiling, Pounding	Root and bark decoction drunk for constipation, abdominal pain, and stomachache. Roots powder used for snake bites.	15, 18, 68
Cactaceae, *Rhipsalis baccifera* (J.S.Muell.) Stearn		FOKP 988	E	Escarpment	R, Br	Boiling	Root and branches decoction drunk as a remedy for gonorrhea.	IR, 15,
Campanulaceae, *Lobelia holstii* Engl.		SAJIT 004798	H	Escarpment, Grassland	R	Boil	Roots decoction drunk for coughs and stomachache for children.	IR, 15,
Canellaceae *Warburgia*	Sokwo (M)	YMM/19/144	T	Escarpment, Woodland	B, L	Boiling, burning, pounding	Bark decoction used for respiratory disorders. Pounding leaves infusion	IR, 15, 32

ugandensis Sprague							drunk for headache. Burning leaves ash licked for allergies.	
Cannabaceae, *Trema orientalis* (L.) Blume	Musakala (L)	FOKP 11562	T	Bushland	L, B	Pounding, Boiling	Leaves juice drunk as a cure for coughs and treatment of pneumonia and bronchitis. Bark decoction drunk for constipation.	70
Capparaceae, *Maerua decumbens* (Brongn.) DeWolf	Chepuluswo (P)	FOKP 1248	S	Bushland	R, L, Fr	Scrubbing, boiling	Root decoction drunk for gonorrhea, syphilis, and as a purgative. Leaves juice used for eye infections. Cools aching body parts by scrubbing with fruit.	15, 34
Capparaceae, *Maerua triphylla* A. Rich.	Chokotwa (M), Chokowa (P)	SAJIT Z0060	S	Bushland	R	Boiling	Boiled root infusion used to cure headache, and as an aphrodisiac.	IR, 15, 73
Capparaceae, *Cadaba farinosa* Forssk.	Arerenion (P), Mvunja-vumo (K)	FOKP 11338	S	Bushland	L	Burning, boiling	Ground leaves used to heal ulcers. Leaf decoction used for the treatment of gonorrhea. Root infusion used against fever.	73
Celastraceae, *Maytenus buchananii* (Loes.) R.Wilczek	Kumwayakhafu (L)	FOKP 11294	S	Thickets	B, R	Boiling	Bark and root decoction drunk for stomachache.	IR

Celastraceae, *Maytenus heterophylla* (Eckl. & Zeyh.) N.Robson	Chirkelwo (M), Likunga (L)	FOKP 11601	S	Escarpment, Highland, Riverine, Thickets	R	boiling	Root decoction used to cure syphilis. Pounded leaf paste used on boils.	IR, 15, 36
Celastraceae, Maytenus obscura (A. Rich.) Cufod.		FOKP 1089	S	Riverine	L	Boiling	Leaf decoction drunk for internal injuries.	15, 34
Celastraceae, *Maytenus senegalensis* (Lam.) Exell	Jirgelwo (M) Akwichanian (P), Muruli (L)	SAJIT 007106	T	Grassland, Riverine	B, R	Boiling	Boiled root infusion used for diarrhea and fever. Leaves decoction drunk for high blood pressure and kidney problems. It's used as blood cleanser.	15, 34, 73, 86, 89
Celastraceae, *Maytenus undata* (Thunb.) Blakelock		SAJIT 4755	T	Highland	B	Boiling	Root decoction drunk for the treatment of syphilis.	68, 90
Celastraceae, *Mystroxylon aethiopicum* (Thunb.) Loes.	Chepkata (P), Muhundui (L)	FOKP 11608	S	Bushland	B	Boiling	Bark decoction drunk for stomachache.	15, 90
Colchicaceae, *Gloriosa superba* L.	Kemagugu (M), Mkalamu (K)	FOKP 11694	C	Bushland	R, L	Boiling	Roots and leaves infusion drunk to secure abortion.	18, 46
Combretaceae, *Combretum apiculatum* Sond.	Leleiya (M)	SAJIT 004849	T	Escarpment, Valley, Grassland	R	Boiling	Leaf decoction used for scorpion bite. Root decoction	IR, 15, 34, 42, 46, 47, 90

							drunk for treatment of diarrhea and snake bite.	
Combretaceae, *Combretum collinum* Fres.	Sheraha (L)	YMM/19/100	T	Grassland	R	Boiling	Root decoction drunk for dysentery.	15, 90
Combretaceae, *Combretum molle* R.Br. ex G.Don	Cheporosto (P), Mukhungula (L)	FOKP 11270	T	Woodland, Escarpment	R, B, L	Boiling, Chewing,	Root and bark decoction drunk for stomachache, intestinal worms, snake bite, fever, leprosy, dysentery, body swelling, T.B. and diarrhea. Leaves chewed for chest pains.	15, 42, 47, 90
Combretaceae, *Terminalia boivinii* Tul.	Goloswa (M), Koloswa (P), Mukhonje (L)	SAJIT Z0055	T	Bushland, Escarpment	B	Chewing, Boiling	Bark chewing is used to treat yellow fever in children. Bark decoction drunk for abdominal pains and body pains and it increases fertility in women.	18, 36, 42, 47
Commelinaceae, *Commelina africana* L.	Nenaitet (M), Nioloniolo (L)	YMM/19/116	H	Valley, Grassland	L, Br	Drying, Boiling,	Branches infusion reduces fever. Leaf juice used as an eye and ear ache remedy. Leaf decoction used in children with colds and coughs.	IR, 67
Commelinaceae, *Commelina benghalensis* L.	Aportotoyon (P), Mpovupovu (K)	FOKP 11415	H	Riverine, Woodland	L, Br	Boiling, Crushing	Leaf decoction drunk for internal injuries. Branches juice used in the treatment of colds, ear ache, and thrush in infants. Leaf decoction drunk for diarrhea.	51, 61, 73, 83, 68

Commelinaceae, *Commelina imberbis* Ehrenb. ex Hassk.	Talamach (P)	YMM/19/125	H	Cultivated	W	Crushing	Whole plant juice reduces fever.	IR, 34
Convolvulaceae, *Ipomoea spathulata* Hallier f.	Kepkerengerwa (M), Talamach (P)	YMM/19/09	S	Bushland,	R	Boiling	Boiled root infusion with milk used as child's tonic. Root decoction drunk for treatment of sterility in women.	IR, 15, 18, 73
Convolvulaceae, *Ipomoea tenuirostris* Choisy	Siliba (M), Tbombolo (L)	FOKP 1885	H	Woodland	L	Crushing	Crushed leaves juice cures rheumatism.	IR, 15, 18
Crassulaceae, *Crassula granvikii* Mildbr.	Shikachi (L)	FOKP 1214	H	Cultivated	Br	Pounding	Pounded branches mixed with water and the infusion drunk for rheumatism.	15, 46
Crassulaceae, *Kalanchoe densiflora* Rolfe	Linyolonyolo (L)	SAJIT 006793	H	Cultivated	R, L	Boiling, Pounding	Pounded leaves applied to wounds. Root and leaf decoction used for abortion.	15, 46, 47, 86
Cucurbitaceae, *Coccinia grandis* (L.) Voigt	Tarmuch (P),	SAJIT 006918	C	Riverine	L	Rubbing	Leaf rubbing on stomach relieves abdominal pains.	15, 73
Cucurbitaceae, *Momordica foetida* Schumach.	Cheserya (M) Lilande (L)	SAJIT 004743	C	Highland, Escarpment	L, R	Crushing, boiling	Crushed leaves mixed with water and used to bathe for cervical cancer treatment. Leaf and root infusion used for abortion and intestinal worm infection. Leaf infusion used as an eye drop.	15, 34, 36, 46, 44, 51, 61, 67

Cucurbitaceae, *Zehneria scabra* Sond.	Cheserya (M)	SAJIT 006799	C	Highland, Escarpment, Valley	R, L	Chewing, boiling	Roots and leaf concoction used to relieve abdominal pains. Pounded leaves used to treat skin rashes. Leaves crushed and administered for coughs.	IR, 15, 42, 71
Dioscoreaceae, *Dioscorea quartiniana* A.Rich.		SAJIT 005067	C	Bushland	R	Boiling	Root infusion used for treatment of fever.	IR, 40
Ebenaceae, *Euclea divinorum* Hiern	Jeptuiya (M), Cheptuya (P)	YMM/19/160	T	Escarpment	R, B, Br, Fr	Boiling, pounding, Chewing	Bark concoction drunk for pneumonia, chest pains and eliminates worms. Roots chewed for toothache and branches used as a toothbrush. Pounded bark paste used for snake bite. Fruits chewed for respiratory disorders.	IR, 11, 15, 18, 36, 42,70, 71, 90
Ebenaceae, *Euclea natalensis* A. DC.	Jeptuiya (M),	YMM/19/085	T	Escarpment	R	Boiling, pounding	Pounded and boiled root decoction eliminates intestinal worms, mouth infections, stomach ulcers, and gonorrhea	IR, 15, 51, 68, 88, 69, 72, 93
Ericaceae, *Agauria salicifolia* (Lam.) Oliv.	Chelemewet (M)	YMM/19/031	T	Highland	B	Boiling	Bark infusion aids digestion	IR

Euphorbiaceae, *Acalypha fruticosa* Forsk.	Mahacha (K), Kaparasamugh (P)	FOKP 11676	S	Valley	R, L, Se	Boiling	Root decoction used for stomachache, gonorrhea, fever, and colds. Leaves pounded and infusion used for diarrhea. Crushed seeds used as a cough mixture.	15, 42, 73, 68
Euphorbiaceae, *Croton dichogamus* Pax	Kerelwo (M), Kekelwa (P)	YMM/19/077	T	Escarpment, Valley, Cultivated	R, L	Boiling	Root decoction used as a treatment for stomachache, chewing leaves relieves colds, chest pains. Leaves ash used for fever.	30, 42, 73
Euphorbiaceae, *Croton macrostachyus* Hochst. ex Delile	Toboswo (M), Musutsu (L)	YMM/19/023	T	Highland, Wooded grassland,	R, B, L	Boiling, Burning	Root decoction used as a remedy for intestinal worms and headache. Burnt leaves used as a cough remedy. Bark decoction drunk for respiratory disorders.	IR, 15, 18, 34, 36, 44, 47, 67, 73
Euphorbiaceae, *Croton megalocarpus* Hutch.	Otonwet (M)	YMM/19/070	T	Woodland	R	Boiling	Decoction of roots used as purgative.	IR, 15, 42
Euphorbiaceae, *Erythrococca bongensis* Pax		FOKP 11430	S	Riverine, Thickets	L	Pounding	Pounded leaves and its decoction used to cure coughs.	15, 92
Euphorbiaceae, *Erythrococca fischeri* Pax	Mboga (P)	FOKP 1234	S	Riverine, Thickets	R	Pounding, Boiling	Root decoction used for infertility and treatment of gonorrhea. Powdered roots used to cure chest pains.	IR, 15, 44, 73

Euphorbiaceae, *Euphorbia heterospina* S.Carter	Gureswa (M)	FOKP 1247	S	Woodland	B	Boiling, Burning	Bark decoction drunk for the treatment of gonorrhea. Burnt leaves used for the treatment of liver diseases.	15, 70
Euphorbiaceae, *Euphorbia obovalifolia* A.Rich.	Kureswa (M), Creswo (P)	SAJIT 006883	T	Highland	Br	Boiling	Boiled branches concoction given to women for stomach pains after childbirth.	15, 73
Euphorbiaceae, *Euphorbia schimperiana* Scheele	Lubondobondwi (L)	SAJIT 005113	H	Grassland, Montane	L, Br	Pounding	Leaf and branches juice drunk as a remedy for coughs and colds.	IR, 15, 70
Euphorbiaceae, *Macaranga kilimandscharica* Pax	Mukaho (L)	SAJIT 006797	T	Highland	R	Boiling	Root decoction drunk as a remedy for coughs. Leaf decoction drunk to cure stomachache.	IR, 15, 70
Euphorbiaceae, *Ricinus communis* L.	Kimonwo (M), Pondon (P) Libono (L),	YMM/19/137	H	Escarpment, Lowland	R, L	Boiling, Pounding	Root decoction used for enhancing appetite, reducing abdominal pains, and treating syphilis. Leaves and stems pounded and juice drunk for ulcers, coughs, stomachache, and diarrhea, also removes placenta after child birth.	IR, 11, 15, 36, 40, 46, 47, 51, 61, 71
Euphorbiaceae, *Tragia brevipes* Pax	Kimelei (M), Chemelei (P)	YMM/19/094	C	Highland, Escarpment, Valley	Br, L, R	Rubbing, Boiling	Rubbing leaves on joints treats rheumatism. Branches and root decoction relieves labor	IR, 15, 18, 34, 40, 42, 44, 67, 71

							pains, diarrhea, and also treats snake bites.	
Geraniaceae, *Pelargonium alchemilloides* (L.) Aiton	Chemendilil (M)	FOKP 1825	H	Wooded, Grassland	L	Pounding	Leaf juice used as an eye drop on sore eyes.	IR, 16
Hypericaceae, *Hypericum quartinianum* A.Rich.	Tabilikwa (M)	YMM/19/033	S	Highland, Escarpment, Valley	Br, L,R	Pounding	Leaves, roots and branches concoction drunk for rheumatism and diarrhea.	IR
Hypericaceae, *Hypericum revolutum* Vahl		SAJIT 004812	S	Forest margins	L, B, Br	Boiling, Pounding	Leaves and branches concoction drunk for diarrhea and rheumatism. Pounded bark paste applied to wounds.	IR, 15,
Lamiaceae, *Ajuga remota* Benth.	Chebonyirar (M), Imbuli-ya-mutakha (L)	FOKP 1713	H	Cultivated	L	Pounding	Leaf infusion drunk as a remedy for fever, malaria, dysentery, high blood pressure, and intestinal worms. Leaves chewed for toothache.	18, 34, 38, 40, 71
Lamiaceae, *Clerodendrum johnstonii* Oliv.	Chebobet (M)	SAJIT 006817	S	Forest margins	L, R	Pounding, Boiling	Leaf juice used as a remedy for toothache. Roots are boiled and drunk for abdominal pains and joint pains.	IR, 34

Lamiaceae, *Clerodendrum myricoides* (Hochst.) R.Br. ex Vatke	Chebobet (M), Shikuma (L)	YMM/19/55	S	Highland, Escarpment, Valley	R	Boiling	Root decoction used as treatment for chest pains, bronchitis, colds, indigestion, sterility, amoebic dysentery, and intestinal worms. Chewing root relieves sore throat, tonsillitis, rheumatism, and malaria.	IR, 34, 39, 40, 42, 36, 47, 61
Lamiaceae, *Fuerstia africana* T.C.E.Fr.	Kipirirwo (M), Pererwo (P) Omwonyo(L)	YMM/19/044	H	Escarpment, Valley, Grassland	L, R, W	Boiling	Root and leaf decoction drunk for malaria and intestinal worms. Whole plant decoction used for treatment for ulcers, respiratory disorders and urinary diseases.	IR, 11, 15, 18, 36, 47, 70, 71
Lamiaceae, *Hoslundia opposita* Vahl	Simbaywa(P), Shikuma (L),	FOKP 11441	S	Bushland, Lowland	R, L	Boiling	Root and leaf decoction used as a cough remedy, fever, colds, blood cleanser, aphrodisiac, amoeba.	11, 15, 61, 73, 86
Lamiaceae, *Leonotis mollissima* Gürke	Kipserere (M), Kipchichin (P), Tsutsuni (L)	SAJIT 004845	H	Highland, Escarpment, Valley	R, L	Boiling, chewing	Root extract used for treatment of wounds, intestinal worms, and dysentery. Chewing leaves relieves stomachache.	IR, 15, 18, 40, 46
Lamiaceae, *Leonotis nepetifolia* (L.) R.Br.	Kachichin (P)	SAJIT 004845	S	Forest margins, Cultivated	L	Crushing	Leaves juice drunk for abdominal pains. Leaf infusion used for fever, coughs, and eye diseases.	15, 46, 61, 73, 86

Lamiaceae, *Leucas calostachys var. fasciculata* (Baker) Sebald	Nechebgwa (M), Cheposorwa (P), Lumitsani (L)	FOKP 11371	H	Grassland, Bushland, Wooded grassland	L	Boiling	Leaf concoction used as an emetic. Leaves juice cures pneumonia and stomachache.	15, 18, 36, 43, 46, 73
Lamiaceae, *Leucas martinicensis* (Jacq.) R.Br.	Shitsunzune shiti (L)	FOKP 11696	H	Bushland	L	Chewing, Boiling	Chewing leaves stops vomiting. Leaf infusion used for eye diseases. Leaf decoction drunk for fever and edema.	15, 70
Lamiaceae, *Ocimum gratissimum* L.	Klachir (M) Omwonyo (L)	YMM/19/101	S	Cultivated	L	Boiling	Leaf decoction drunk for stomachache and constipation for children.	IR, 15, 42, 47, 61, 67, 68, 71, 86
Lamiaceae, *Ocimum kilimandscharicum* Gürke	Mwonyi (L)	SAJIT 004746	S	Escarpment, Riverine, Grassland	L	Crushing, Boiling	Leaves infusion cures colds. Leaf juice applied on insect bites.	15, 40, 43, 51
Lamiaceae, *Ocimum suave* Willd.	Chesimia (M), Taiya (P), Kirumbasi (K)	YMM/19/018	H	Grassland, Riverine	L	Rubbing, boiling	Sniffing leaves treats blocked nostrils. Leaf infusion used for abdominal pain, sore eyes, and coughs.	IR, 15, 73, 86
Lamiaceae, *Plectranthus barbatus* Andrews	Angurwo (M), Shilokho (L)	FOKP 11368	S	Escarpment, Grassland	R, L	Crushing	Leaf decoction used for fever and malaria. Root decoction used for stomach ache.	15, 34, 47, 61, 71, 89, 90

Lamiaceae, *Plectranthus caninus* Roth		FOKP 11343	H	Cultivated	L	Chewing	Chewing leaves relieves toothache. Root infusion drunk as a cough syrup.	IR, 15
Lamiaceae, *Plectranthus laxiflorus* Benth.	Lonwo (M)	YMM/19/040	H	Valley	R, L	Pounding	Pounded leaves used as a remedy for intestinal worms. Roots chewed for sore throat.	IR, 86
Lamiaceae, *Satureja abyssinica* (Benth.) Briq.		FOKP 1085	H	Escarpment	L, Br	Boiling, Burning	Ash from burned leaves and branches used for coughs. Leaves used for indigestion.	18, 15, 68
Lamiaceae, *Tinnea aethiopica* Kotschy ex Hook.f.		FOKP 11692	S	Thickets, Forest margins	R, L	Pounding	Pounded leaves juice used for conjunctivitis and abdominal pains.	15, 68
Leguminosae, *Acacia brevispica* Harms	Kiptare (M), Kiptara (P),	SAJIT Z0058	T	Bushland, Thickets	R, L	Boiling, Pounding	Roots infusion drunk as a cure for intestinal worms and fever. Root decoction used as an aphrodisiac	11, 15, 18, 47, 51, 73
Leguminosae, *Acacia reficiens* Wawra	Leina (M), Panyarit (P)	YMM/19/072	T	Highland, Valley	R	Boiling	Root decoction used against stomachache and also used as an aphrodisiac.	IR, 47
Leguminosae, *Acacia hockii* De Wild.	Chuina (M), Chooh (P), Liwabwa (L),	YMM/19/079	T	Highland, Valley	B, R	Boiling	Root decoction used for abdominal pains. Women with gynecological problems use bark decoction.	IR, 11, 15, 18, 32, 40, 47, 61, 70, 73

Leguminosae, *Acacia lahai* Benth.	Telak (M)	Mbuni 005	T	Highland, Woodland	B, R	Boiling	Root and bark concoction drunk for the treatment of measles.	11, 15, 18, 32, 36, 47
Leguminosae, *Acacia mellifera* (M.Vahl) Benth.	Belel (M), Talamong (P),	SAJIT Z0064	T	Escarpment, Woodland	B, R	Boiling	Bark and root concoction drunk for syphilis, sterility, pneumonia, malaria, coughs, and chest pains.	11, 15, 18, 30, 34, 42,68, 47, 51,
Leguminosae, *Acacia nilotica* (L.) Delile	Ngobgwa (M), Kopokwo (P)	YMM/19/148	T	Escarpment, Valley	B, L, R	Boiling	Bark infusion drunk for indigestion and is used as an eye medicine. A concoction of leaves, bark, and roots are used to treat chest pain, gonorrhea and as an aphrodisiac.	IR, 11, 18, 30, 42, 47, 51, 68, 73
Leguminosae, *Acacia tortilis* subsp. *spirocarpa* (A.Rich.) Brenan	Ses (P)	FOKP 1249	T	Escarpment,	R, B	Boiling	Bark decoction drunk for stomach ache. Powdered roots used for skin diseases.	11, 30, 42, 47, 51, 73
Leguminosae, *Caesalpinia decapetala* (Roth) Alston	Gakarkaren (P), Lunani (L)	FOKP 1297	S	Bushland, Forest margins	R, L	Pounding	Pounded leaves infusion drunk for epilepsy. Roots decoction drunk for diarrhea.	15, 71, 90
Leguminosae, *Cassia abbreviata* Oliv.		SAJIT 004734	S	Bushland	R, B	Boiling	Roots and bark concoction drunk to cure malaria, stomachache, gonorrhea, and chest pains.	15, 83, 87, 90, 91

Leguminosae, *Crotalaria axilaris* Aiton		FOKP 11613	S	Highland	L	Rubbing	Leaves applied to the eyes to cure conjunctivitis.	34
Leguminosae, *Crotalaria brevidens* Benth.		FOKP 11482	S	Grassland, Bushland	L	Boiling	Leaves used as a remedy for stomachache and body swellings.	IR, 15, 70
Leguminosae, *Crotalaria deserticola* Baker f.	Kipgurgur (M)	FOKP 11483	S	Wooded grassland	L	Pounding	Pounded leaves used for wounds.	15, 51
Leguminosae, *Crotalaria incana* L.	Kimira (M), Lisilinyende (L)	FOKP 11347	S	Escarpment	R, L	Pounding, Boiling	Pounded leaf paste applied for treatment of wounds. Root decoction drunk to position the baby's placenta and for stomach upsets. Used as a vegetable.	11, 15, 18, 46
Leguminosae, *Dalbergia lactea* Vatke	Rokokon (P)	SAJIT 006880	H	Riverine	L, R	Boiling	Roots and leaves used for abdominal pains.	71, 73
Leguminosae, *Desmodium repandum* (Vahl) DC.		FOKP 11525	H	Highland	R, L	Boiling	Roots and leaf decoction used to treat abdominal pains in children.	43
Leguminosae, *Entanda abyssinica* A. Rich.	Masembaa (M) Musembe (L)	SAJIT 006851	T	Woodland	L, B	Boiling	Decoction of roots drunk for rheumatism. Leaf and bark concoction drunk for stomachache and Oedema.	15, 36, 40, 43, 44, 46, 61

Leguminosae, *Entada leptostachya* Harms		SAJIT 004842	C	Bushland	R	Boiling	Roots concoction drunk for the treatment of TB.	15, 42
Leguminosae, *Erythrina abyssinica* DC.	Gorgorwa (P), Korkorwo (M) Omurembe (L),	YMM/19/088	T	Woodland, Highland	R, B	Boiling	Root infusion used to stimulate evacuation of bowels. Boiled bark administered for mumps, anemia, fever, liver inflammation.	IR, 11, 15, 18, 34 36, 40, 42, 46, 47, 61, 73
Leguminosae, *Glycine wrightii* Lopez	Manguwian (P),	FOKP 11450	H	Cultivated	L	Boiling	Leaf decoction drunk to stop bleeding as a result of a miscarriage.	15, 44, 46, 73
Leguminosae, *Indigofera ambelacensis* Schweinf.	Sargellat (M)	FOKP 11475	H	Grassland	R	Chewing	Roots are chewed for heart diseases.	IR
Leguminosae, *Indigofera arrecta* A.Rich.	Perkeletwo (M), Chesutow (P), Lweyu (L)	YMM/19/049	H	Escarpment, Valley	R, L	Pounding, Boiling	Root decoction used for the treatment of stomach ache, heart, and kidney diseases. Leaves are used for toothache.	IR, 11, 15, 18, 36, 46, 47, 61, 70, 71, 73, 86
Leguminosae, *Indigofera trita* L.f.	Sarkelat (M)	YMM/19/122	H	Escarpment, Valley	L, R	Pounding, Crushing	Leaves and roots pounded, mixed with water, and the infusion used to cure headache and stomach ache.	IR, 15, 68
Leguminosae, *Pterolobium*		FOKP 1265	S	Bushland	R, L	Boiling	Roots decoction drunk for the treatment of stomach ache. Leaves decoction used for the	IR, 15,

stellatum (Forssk.) Brenan							treatment of TB and chest pains	
Leguminosae, *Rhynchosia hirta* (Andrews) Meikle & Verdc.		SAJIT 006841	L		R, L	Boiling	Root decoction drunk for quick and painless delivery and removal of a retained placenta. Roots and leaves used for stomach ache.	61
Leguminosae, *Senna didymobotrya* (Fresen.) H.S.Irwin & Barneby	Senetwet (M), Lubino (L)	YMM/19/127	S	Escarpment, Valley	R, Br, L	Pounding	Leaves decoction used to cure diarrhea, backaches in women, and stomachache. Roots and branches concoction used to cure malaria and headache.	IR, 36, 42, 44, 47, 61, 67, 71, 73, 86, 90
Leguminosae, *Senna septemtrionalis* (Viv.) H.S.Irwin & Barneby	Mugusa (L)	FOKP 1903	S	Cultivated	R, L, Fr	Boiling	Leaf decoction drunk for removal of a retained placenta. Pounded fruits and roots infusion drunk for purgative.	IR, 15, 51
Leguminosae, *Tamarindus indica* L.	Aron (M), Oron P),	YMM/19/155	T	Escarpment	R, Fr, L	Boiling	Branches and leaves boiled in water and drunk for diarrhea, dysentery, measles and chickenpox. Bark and root concoction drunk for coughs, fevers, malaria, asthma, leprosy, ulcers, liver disease, and rheumatism.	IR, 15, 18, 51, 61, 70, 78

Leguminosae, *Trifolium rueppellianum* Fresen.	Kwishihelechero (L)	FOKP 1128	H	Grassland	Br	Boiling	Infusion of pounded branches given to children with measles and intestinal worms.	15, 46
Loganiaceae, *Strychnos henningsii* Gilg	Chapkamkam (P)	SAJIT Z0035	T	Woodland	R, B, L	Boiling	Decoction of roots drunk for chest pains and backache. Root, bark and leaf concoction used for malaria.	IR
Loranthaceae, *Agelanthus sansibarensis* (Engl.) Polhill & Wiens	Tworop sitet (M)	YMM/19/136	S	Highland, Escarpment	Br, L	Burning, Pounding	Leaves and branches burnt and ash pound and licked as a remedy for headache.	IR, 15,
Loranthaceae, *Englerina woodfordioides* (Schweinf.) Balle	Mondoywo tobongwo (M), Sagorgetia (P)	SAJIT 007053	S	Highland, Escarpment, Valley	L, B	Boiling	Decoction of bark and leaves used to treat liver and spleen diseases. Heated twigs placed on the chest to treat pneumonia.	IR, 18, 15, 70
Loranthaceae, *Erianthemum dregei* (Eckl. & Zeyh.) Tiegh.		SAJIT 004847	S	Forest edges, Bushland	B	Pounding	Pounded bark applied on snake bite wounds.	IR, 15,
Loranthaceae, *Phragmanthera dschallensis* (Engl.) M.G.Gilbert	Tworop tobongwa (M), Shikulali (L)	SAJIT 006865	S	Highland, Escarpment, Valley, Wooded, rassland	Br, L	Boiling	Branches and leaves decoction drunk for chest pain and pneumonia.	36

Loranthaceae, *Phragmanthera usuiensis* (Oliv.) M.G.Gilbert	Sagorgetia, Torwa ngurwa (M)	FOKP 11323	S	Highland, Escarpment, Valley, Montane	Br, L,	Burning	Branches and leaf ash licked and used to treat colds, paralysis, stroke, and gout.	IR, 16, 36
Malvaceae, *Dombeya burgessiae* Gerrard ex Harv.	Mukusa (L)	SAJIT 004764	T	Riverine, Bushland	R, B, L	Chewing, boiling	Bark is chewed as an aphrodisiac. Root infusion drunk for stomach pains. Leaf decoction drunk to remove retained placenta.	15, 46
Malvaceae, *Dombeya rotundifolia* (Hochst.) Planch.	Mogonja (M), Mugeriswa (P)	YMM/19/067	T	Highland	R	Boiling	A decoction of roots drunk for rheumatism. Root juice soaked in water used to treat diarrhea in children.	IR, 18, 86, 90
Malvaceae, *Dombeya torrida* (J.F.Gmel.) Bamps	Borowo (M)	YMM/19/095	T	Highland	B	Boiling	A decoction of bark used to treat indigestion allergies and joint pains.	IR, 15, 16, 36, 70
Malvaceae, *Hibiscus fuscus* Garcke	Cheptelia (M), Pkapuyan (P)	YMM/19/118	H	Cultivated	R, L	Boiling, Chewing	Roots chewed to cure coughs and as an aphrodisiac. Leaves also chewed for chest diseases	IR, 15, 43, 46, 47, 71
Malvaceae, *Grewia similis* K.Schum.		FOKP 1025	T	Woodland	B	Pounding, Boiling	Pounded bark applied on wounds and snake bites. Bark decoction drunk for sore throat and constipation.	51, 90
Malvaceae, *Pavonia urens* Cav.	Pelpany (P)	FOKP 11403	S	Riverine, Forest margins	R	Boiling	Root decoction drunk for abdominal pains.	34

Malvaceae, *Sida acuta* Burm.f.	Korkoriet (P),	FOKP 11509	H	Cultivated	L	Crushing	Leaves crushed and extract applied on cuts.	15, 90
Malvaceae, *Sida tenuicarpa* Vollesen	Amekunyan (P)	SAJIT 007066	S	Highland, Grassland	R	Boiling, Chewing	Roots chewed for the treatment of sore throat and to remove retained placenta after birth.	15, 44, 47, 70
Malvaceae, *Triumfetta brachyceras* K.Schum.	Shambamaches e (L)	YMM/19/102	S	Forest margins	L	Crushing	Leaves crushed and paste applied to the boil to make it burst within a few days.	IR
Malvaceae, *Triumfetta cordifolia* A.Rich.		FOKP 11333	S	Montane forest	L	Boiling	Leaf decoction used for the treatment of hepatic diseases.	IR
Melastomataceae, *Dissotis speciosa* Taub.		YMM/19/112	S	Escarpment	L	Chewing, Pounding	Pounded leaves chewed for coughs and diarrhea.	IR, 43
Meliaceae, *Ekebergia capensis* (Fresen. A. Rich)	Kerbut (M)	SAJIT 005079	T	Highland, Escarpment, Riverine	R, B	Chewing, Boiling	Bark decoction drunk for the skin rashes, skin cancer, TB and backache. Roots are chewed as an expectorant.	IR, 18, 36, 40, 43, 67, 90
Meliaceae, *Lepidotrichillia volkensii* (Gurke) Leroy	Tsisiema (L)	FOKP 1068	T	Forest margins	L	Pounding	Leaf pound and infusion assists children with a stomachache.	16
Melianthaceae, *Bersama abyssinica* Fresen.	Kipset (M),	SAJIT 007049	T	Montane forest	R, B,	Pounding, Boiling	Root decoction drunk for epilepsy, cancer and infertility in women and men. Leaves, branches and bark are chewed	18, 36, 46, 67

					L, Br		as an aphrodisiac and for coughs.	
Menispermaceae, *Cissampelos pareira* L.	Malutiatito (P)	FOKP 11289	C	Bushland	R, L	Pounding, Burning	Burnt roots and leaves ash applied on wounds. Roots and leaves chewed for stomachache and hypertension. Powdered roots used as a remedy for coughs, sore throat, colds and snake bite.	IR, 36, 76, 68
Monimiaceae, *Xymalos monospora* (Harv.) Baill.		SAJIT 004857	T	Woodland	Fr	Pounding	Eating fruits eliminates roundworm and tapeworm, and they are used as a remedy for chest pains.	15, 90
Moraceae, *Ficus glumosa* Delile		SAJIT 006836	T	Woodland	B	Boiling	Bark infusion drunk for stomachache and diarrhea.	IR
Moraceae, *Ficus natalensis* Hochst.	Lutoto (L)	FOKP 1032	T	Woodland	B	Boiling	Bark decoction drunk for influenza and gonorrhea.	15, 61, 90
Moraceae, *Ficus sur* Forssk.	Poyotwo (P)	SAJIT 006849	T	Riverine, bushland	R, B	Boiling	A root decoction is taken as a cough remedy. Bark infusion drunk to treat stomachache and diarrhea.	15, 36, 67 72, 91
Moraceae, *Ficus vasta* Forssk.	Beriotwa (M), Cheptokelat (P)	YMM/19/146	T	Highland	B	Boiling	Bark decoction drunk as an astringent and Swelling of the body.	11, 15, 18

Myrtaceae, *Psidium guajava* L.	Mapera (M), Lipera (L)	FOKP 11471	T	Bushland	L	Boiling	Leaf decoction drunk for jaundice and coughs. Tender leaves are chewed for colic pains.	IR, 47, 51 61, 70, 67, 90
Myrtaceae, *Syzygium guineense* (Willd.) DC.	Lamaiwo (M), Cheptimanwa (P),	SAJIT 005086	T	Wooded grassland, Riverine	B, Fr	Pounding	Pounded bark treats stomachache, abdominal pains, heartburn and as an aphrodisiac. Fruits are eaten as a remedy for dysentery.	40, 44, 47, 70, 90
Myrtaceae, *Syzygium cordatum* Hochst. ex Krauss	Seberwa (M)	FOKP 1267	T	Riverine	R, B	Boiling	Roots and bark boiled and decoction drunk for indigestion, coughs, abdominal pains, stomachache, and gonorrhea.	18, 36, 61, 90
Nyctaginaceae, *Commicarpus plumbagineus* (Cav.) Standl.	Tanagit (M), Lopuchin (P),	FOKP 1250	H	Riverine, Woodland	L	Crushing	Crushed leaves applied on cuts.	IR, 18
Oleaceae, *Jasminum fluminense* Vell.	Kolion (P)	SAJIT 00680	S	Bushland	R	Rubbing	Roots powder used for snake bite. A decoction of root drunk for chronic coughs and chest pain.	IR, 81
Oleaceae, Olea capensis L.	Pekeriondet (M)	YMM/19/075	T	Woodland	B	Soaking Boiling	Bark decoction used for coughs and malaria.	IR, 15, 36, 70
Oleaceae, *Olea europaea* L.	Yemit (M), Yemut (P)	SAJIT 004756	T	Escarpment, Highland	B, R, L	Boiling	Root and bark decoction used to treat malaria, tapeworms and itchy rashes. Leaf decoction drunk for hepatic	IR, 11, 15, 30, 34, 36, 61, 70, 90

							diseases and treatment of amoeba.	
Orchidaceae, *Ansellia africana* Lindl.	Sokorokel (M)	FOKP 11307	H	Escarpment	Br	Heating	Branches heated over a fire and then the juice is squeezed into the ears as ear drop.	IR, 15, 78, 90
Passifloraceae, *Adenia cissampeloides* (Planch. ex Hook.) Harms		SAJIT 004724	C	Highland	L, Br	Crushing	Crushed leaves and branches applied on wounds.	IR, 15
Pedaliaceae, *Sesamum angolense* Welw.		FOKP 1039	S	Grassland	R, L	Pounding	Pounded leaves and roots used to treat measles.	IR
Pedaliaceae, *Sesamum calycinum* Welw.		FOKP 1040	H	Grassland	R, L, Se	Pounding	Pounded root and leaf decoction are drunk for stomach ache. Seed oil treats ringworm.	IR
Peraceae, *Clutia abyssinica* Jaub. & Spach	Chekelel (M), Sambukwe (L)	SAJIT 007042	S	Highland, Escarpment, Valley	R	Boiling	Root decoction drunk for headache, malaria, stomachache, influenza, indigestion, and liver pains.	IR, 11, 18, 39 40, 86
Penaeaceae, *Olinia rochetiana* A.Juss.	Nerkwo (M),	FOKP 1839	T	Highland	R, B	Boiling, chewing	Root decoction used against coughs and fever. Bark decoction used to treat bronchitis, indigestion, rheumatism, and tapeworms.	IR, 36

Phyllanthaceae, *Phyllanthus fischeri* Pax		SAJIT 004778	S	Riverine	R, L	Boiling	Leaf and root decoction drunk for gynecological problems.	84
Phytolaccaceae, *Phytolacca decandra* L.	Kipsugotit (M), Libokho (L)	SAJIT 006884	S	Highland, Valley	R, L	Boiling, Pounding	Root decoction used for roundworm, tapeworm, and syphilis. Pounded leaves mixed with water and used to bathe for skin rashes.	IR, 15, 18, 61, 67
Piperaceae, *Piper capense* L.f.	Chepnonet (M)	YMM/19/032	H	Highland	R, L, Se	Boiling	Root decoction used as an anthelmintic. Leaves pounded and infusion used for diarrhea. Crushed seeds used as a cough mixture.	IR, 43, 44, 90
Pittosporaceae, *Pittosporum lanatum* Hutch. & E.A.Bruce	Chemaroryet (M)	FOKP 11391	T	Escarpment	B	Pounding, Boiling	Pounded bark is taken with water as a remedy for malaria. Bark decoction used as an emetic.	15, 16, 18
Pittosporaceae, *Pittosporum mannii* Hook.f.	Mmonyia (L)	SAJIT Z0026	S	Valley	B, L	Boiling	Leaf and bark decoction drunk for malaria and to stop bleeding.	15, 46, 47
Pittosporaceae, *Pittosporum viridiflorum* Sims	Jemnoa (M), Chelewo (P)	SAJIT 007050	T	Highland Wooded grassland	B	Boiling	A decoction from bark drunk for chest pains, malaria, amoeba, fever, loss of appetite, emetic and constipation.	IR, 15, 90
Plantaginaceae, *Plantago palmata* Hook.f.		FOKP 1065	H	Montane	L, R	Boiling, Crushing	Leaf and root decoction used for liver diseases. Leaves are crushed, mixed with water and	15, 40, 61

							used to bathe children to eradicate skin rashes.	
Podocarpaceae, *Podocarpus falcatus* (Thunb.) Endl.	Benet (M)	FOKP 1797	T	Highland, Woodland	B	Boiling	A decoction of bark drunk for the treatment of stomach ache and hypertension.	36, 70, 73
Polygalaceae, *Polygala sphenoptera* Fresen.		SAJIT 005100	H	Grassland	R	Pounding	Pounded roots used for the treatment of coughs and as an aphrodisiac.	IR, 15
Polygalaceae, *Polygonum salicifolium* Brouss. ex Willd.	Burika (L)	FOKP 1129	H	Grassland	L	Burning	Burnt leaves ash used to cure sore throat and tonsillitis. Leaf powder rubbed on the skin for skin diseases.	16
Polygalaceae, *Polygonum setosulum* A.Rich.		FOKP 1734	H	Montane	W	Boiling	Boiled whole plant used to cure malaria. The whole plant pounded and tied around the wound.	61
Polygalaceae, *Rumex usambarensis* (Dammer) Dammer	Kibolongbong (M)	FOKP 11565	H	Riverine	L, R, W	Pounding, Boiling	Pounded leaves used for coughs. A decoction of the whole plant used for smallpox. Root decoction used to treat scabies.	86
Primulaceae, *Maesa lanceolata* Forssk.	Ribotio (M), Mushebeshebe (L)	SAJIT 005138	T	Highland	Fr, R	Pounding, Boiling	Roots decoction used to lower abdominal pains during pregnancy and kidney diseases. Root decoction used	IR, 30, 36, 61

							for snake bite. Fruits are eaten for heartburn.	
Primulaceae, *Myrsine africana* L.	Segatet (M)	SAJIT 004801	S	Highland	B, Fr, Se	Pounding, Eating	Eating fruits and seeds eliminate roundworm and tapeworm and also used as a remedy for respiratory disorders.	46, 70
Primulaceae, *Rapanea melanophloeos* (L.) Mez	Sitotwa (M)	SAJIT 007048	T	Highland, Moorlands	Se	Pounding	Pounded seeds cause the removal of intestinal worms.	IR, 36, 90
Proteaceae, *Faurea saligna* Harv.	Murkwa (M), Chepkonyeng (P)	FOKP 1092	T	Highland, Woodland, Grassland	B	Boiling	Bark decoction drunk for diarrhea and indigestion. A decoction of bark relieves pains during monthly periods.	IR, 11, 90
Ranunculaceae, *Clematis brachiata* Thunb.	Pising (M), Lunyili (L)	FOKP 11277	C	Bushland, Grassland	R, L, Fl	Boiling, sniffing	Roots and leaves decoction is used as a remedy for malaria, colds, headache. Sniffing flowers clears stuffy nose.	90
Ranunculaceae, *Clematis simensis* Fresen.	Bisung'wa (M), Shiraha (L)	FOKP 993	C	Bushland	R, L	Boiling, chewing	Root and leaves decoction drunk for the treatment of malaria.	16, 18
Ranunculaceae, *Ranunculus multifidus* Forssk.	Baiwandab tarit (M)	FOKP 1207	H	Upland	R	Pounding	Pounded root decoction used as an emetic.	16, 18, 93

Ranunculaceae, *Thalictrum rhynchocarpum* Dillon & A. Rich	Kipsuruny (M)	SAJIT 005057	H	Upland	R, L	Boiling	Leaf juice used to treat hepatic diseases. Root decoction used as an expectorant.	16, 18
Resedaceae, *Caylusea abyssinica* (Fresen.) Fisch. & C.A.Mey.	Jeptabilikwa (M)	FOKP 11511	H	Grassland	W, R	Burning, Boiling	Burnt ash from whole plant and root decoction used as a remedy for abdominal pains and intestinal worms.	IR, 18
Rhamnaceae, *Scutia myrtina* (Burm.f.) Kurz	Simboiywo (M) Kitusutut (P)	SAJIT 005064	H	Riverine	R, L	Boiling	Root and leaves decoction used to eradicate intestinal worms. Bark boiled and drunk for respiratory disorders.	18, 70, 68
Rhamnaceae, *Rhamnus prinoides* L' Her'it	Kosisit (M)	SAJIT 007052	T	Bushland	R, B, Br	Boiling	Root, bark and branches decoction used for gonorrhea and abdominal pains.	IR, 18, 30, 40 70, 67
Rhamnaceae, *Rhamnus staddo* A.Rich.	Kipsur (M), Chepsireu (P)	SAJIT 007070	S	Bushland	R, B	Boiling	Root decoction drunk to cure venereal diseases. Bark decoction drunk for colds.	IR, 15, 18, 30
Rhamnaceae, *Ziziphus mauritiana* Lam.	Tilomwa (M), Tolomwo (P), Mkunazi (K)	YMM/19/139	S	Riverine, Bushland	R, B	Boiling, Chewing	A root infusion is drunk for Tuberculosis, indigestion, and dysentery. The bark is chewed raw as a remedy for abdominal pains.	IR, 18, 42, 73
Rhamnaceae, *Ziziphus mucronata* Willd.	Nonoiywa (M), Tirokwo (P),	FOKP 11334	T	Riverine, Woodland,	B, R	Boiling	Boiled bark infusion used against stomachache. Roots powder used for snake bite.	IR, 15, 18, 61, 68,69 , 90

Rosaceae, *Hagenia abyssinica* (Bruce ex Steud.) J.F.Gmel.		SAJIT 4854	T	Woodland	R, B, Fl	Boiling, Pounding	Roots decoction used for malaria. Pounded bark used as a remedy for diarrhea and stomachache. Pounded flowers used for removal of tapeworms.	15, 16, 18, 67
Rosaceae, *Prunus africana* (Hook.f.) Kalkman	Tendwo (M)	SAJIT 006869	T	Woodland, Highland	B, L	Pounding	Pounded bark drunk for stomachache and prostate cancer. Leaf infusion used to treat malaria.	11, 15, 34, 36, 40, 43, 44, 61 70, 90
Rosaceae, *Rubus steudneri* Schweinf.	Momonwa (M)	SAJIT 006811	C	Bushland, Escarpment	R, L	Boiling	Root decoction drunk as a remedy for coughs. Leaf decoction drunk as a remedy for abdominal pains.	IR, 18, 40, 67
Rubiaceae, *Gardenia volkensii* K.Schum.	Magilion (M), Kopulwo (P)	SAJIT 006878	S	Riverine Woodland, Valley	Fr	Boiling	Fruit decoction used to treat malaria and fruit infusion used as an emetic and for skin cancer.	11, 15, 18, 44, 73, 68
Rubiaceae, *Keetia gueinzii* (Sond.) Bridson	Mukoye (L)	SAJIT 005066	C	Bushland, Riverline	R, B	Boiling	Root and bark decoction drunk for diarrhea and joint pains.	15, 70, 92
Rubiaceae, *Oldenlandia Herbacea* (L.) Roxb.	Lambula (L)	FOKP 11315	H	Escarpment	R	Boiling	Root decoction used for syphilis and as medicine for sore throat.	15, 34
Rubiaceae, *Oldenlandia monanthos*		SAJIT 006820	H	Grassland	W	Boiling	Whole plant decoction used for the treatment of hemorrhoids.	15, 34

(Hochst. ex A.Rich.) Hiern								
Rubiaceae, Pavetta abyssinica Fresen.	Jeptabirirwa (M), Chemchirpoma sop (P)	SAJIT 005069	S	Escarpment, Riverine	R, B	Boiling, Crushing	Boiled root decoction drunk for indigestion. Bark crushed and administered as a purgative.	11, 15, 18, 36
Rubiaceae, Pentas longiflora Oliv.	Jepkole (M)	SAJIT 007075	H	Wooded grassland	R	Boiling	A decoction of roots used to cure tapeworm, skin rashes, and malaria.	18, 36, 40, 71
Rubiaceae, *Pentas pubiflora* S.Moore	Chebirirgorok (M)	FOKP 11290	S	Bushland	R	Boiling	A root decoction is drunk as a purgative.	IR, 18
Rubiaceae, Pentas zanzibarica (Klotzsch) Vatke	Chebirirgorok (M)	FOKP 11620	S	Grassland	R, L	Boiling	Root and leaf decoction taken as a remedy for gonorrhea and syphilis.	15, 70
Rubiaceae, *Psychotria kirkii* Hiern	Chemakol (P)	FOKP 11298	S	Bushland, Wooded grassland	R, Fr	Boiling, Crushing	Crushed fruits and boiled roots drunk as a purgative.	70
Rubiaceae, *Psychotria mahonii* var. *puberula* (E.M.A.Petit) Verdc.	Munakuveka (L)	SAJIT 007088	T	Grassland, Bushland	R, Br	Boiling	Root and branches decoction used to gurgle in the mouth for sore throat.	15, 70

Rubiaceae, *Rubia cordifolia* L.	Psalua (P) Olunda nguvo(L)	YMM/19/047	C	Forest margins, Riverine, Bushland	R, L	Boiling, Pounding	Roots and leaves pounded used for treatment of stomach ache and diarrhea. Root decoction used as a remedy for chest pain.	IR, 15, 18, 36, 39, 40, 47, 71
Rubiaceae, *Spermacoce princeae* (K.Schum.) Verdc. .	Kisunda shikuu(L)	SAJIT 006926	H	Bushland	W	Pounding	Whole plant pounded and used for skin diseases.	38, 40, 44, 47
Rubiaceae, *Vangueria apiculata* K.Schum.	Tabiririwo (M), Komolwo(P), Shikomoli (L)	SAJIT 005061	T	Thickets, Riverine, Escarpment	L, R, Br	Chewing, Boiling, Burning	Chewing leaves cures stomachache. Root decoction used for intestinal worms. Ash from branches is licked for tuberculosis.	11, 15, 36, 61
Rubiaceae, *Vangueria infausta* Burch.	Tabiririwo (M), Komolwo(P)	SAJIT 006853	S	Bushland	R	Boiling	Root decoction used for coughs, gonorrhea, infertility and erectile dysfunction.	68, 69, 91, 92
Rubiaceae, *Vangueria madagascariensis* J.F.Gmel.	Komolwo(P)	Mbuni 751	T	Escarpment, Wooded grassland, Bushland	R, B	Boiling, Pounding	Infusion from pounded bark drunk for treating malaria. Root decoction drunk for intestinal worms.	15, 70, 73
Rutaceae, *Clausena anisata* (Willd.) Hook.f. ex Benth.	Cheboinoiywa (M), Kisimbari (L)	FOKP 11460	S	Bushland, Forest margins	R, B, Br	Pounding, Boiling	Pounded roots used for malaria, headache, emetic, coughs, fever, and indigestion. Branches used to cure toothache. Root decoction	IR, 15, 18, 36, 46 ,67, 68

							drunk for the treatment of kidney and heart diseases.	
Rutaceae, *Teclea nobilis* Delile	Lugumwa (M), Chemchir (P), Mutaro (L)	FOKP 11637	T	Woodland	R, L	Boiling	Root infusion used as emetic, TB, children coughs, and fevers. Boiled infusion of leaves used for headache. Bark infusion used as body cleanser.	15, 18, 36, 47, 61
Rutaceae, *Toddalia asiatica* (L.) Lam.	Kipkeres (M), Katamwa (P),	SAJIT 005074	L	Forest margins	R, Fr, B	Chewing, Boiling	Roots and fruits chewed for coughs, colds, back pains, malaria, headache, indigestion, and toothache. Bark and leaf decoction taken for stomach ache and kidney problems. Chewing fruit for respiratory disorders.	IR, 15, 18, 36, 38, 40, 43, 46, 70, 77, 90
Rutaceae, *Teclea simplicifolia* (Engl.) I. Verd.	Kuriot (M)	YMM/19/150	T	Bushland	B	Boiling	Bark decoction used for malaria, fever and oral trash.	IR, 70
Rutaceae, *Zanthoxylum chalybeum* Engl.	Songoiywa (M), Songowowo (P)	YMM/19/105	T	Bushland, Escarpment	R, B,L	Boiling, Chewing, Pounding	Pounded roots drunk for stomachache, sore throat, and fever. Bark decoction is used for colds, coughs, and malaria. The bark is chewed for toothache. Leaves used for snake bite.	IR, 11, 15, 18, 30, 61, 70, 79, 90

Salicaceae, *Dovyalis abyssinica* (A.Rich.) Warb.	Mindillilwo (M)	SAJIT 007057	T	Woodland	L, B, R	Pounding, Boiling	Leaves infusion drunk for indigestion. Roots decoction drunk for gonorrhea stomachache joint pains and fever.	15, 18, 40, 70
Salicaceae, *Dovyalis macrocalyx* (Oliv.) Warb.	Kapchebinin (M) Likunga (L)	SAJIT 007079	S	Highland	R, B	Boiling	Bark decoction drunk for syphilis. Root decoction is taken for headache and ulcers.	15, 44, 47, 70
Salicaceae, *Flacourtia indica* (Burm.f.) Merr.	Tungururwo (M), Tingoswa (P)	FOKP 11288	T	Forest margins	R, L	Boiling	Leaves infusion drunk for asthma. Root decoction used for indigestion, diarrhea, gonorrhea, infertility, and stomachache.	IR, 18, 36, 70
Salvadoraceae *Salvadora persica* L.	Asiokonion (P), Chekowo (M)	FOKP 1253	T	Riverine, Woodland	R, B	Boiling	Root decoction drunk for malaria, dizziness, TB, and chest pains. Bark decoction is taken for fever and colds.	11, 30, 70, 73, 68
Santalaceae, *Osyris lanceolata* Hochst. & Steud.	Jemokabil (M), Mornorwo (P)	SAJIT 004835	S	Escarpment, Bushland, Thickets	R, B, L	Pounding, Boiling	Pounded leaves are drunk for fever, diarrhea and stomachache.	15, IR, 18, 73, 86, 90
Santalaceae, *Viscum fischeri* Engl.	Tworop tabirikwa (M)	YMM/19/092	S	Bushland	B	Heating	Heated branches used for chest pains and pneumonia.	IR
Santalaceae, *Viscum*		SAJIT 006852	P	Woodland	Br	Heating	Heated branches used on the chest of a person with pneumonia.	IR, 70

tuberculatum A.Rich.								
Sapindaceae, *Allophylus abyssinicus* (Hochst.) Radlk.	Chepkokai (P), Lusasari (L)	SAJIT 006927	T	Riverine	R	Pounding	Roots crushed and applied where there are ring worms.	67, 68
Sapindaceae, *Cardiospermum grandiflorum* Sw.	Burili (L)	YMM/19/120	C	Upland	L	Pounding	Infusion of pounded fresh leaves cures fever and ringworm.	IR, 90
Sapindaceae, *Deinbollia borbonica* Scheff.	Musana (L)	FOKP 1068	T	Cultivated	R	Chewing, Boiling	Roots decoction used for stomachache. Roots are chewed for toothache.	15, 68
Sapindaceae, *Dodonaea viscosa* (L.) Jacq.	Tabilikwa (M), Topolokwo (P), Muendu (L),	SAJIT 004844	S	Escarpment, Bushland	R, B, L, Br	Boiling, Pounding	Boiled roots and bark concoction stimulates milk in women after childbirth and is used for respiratory disorders. Pounded leaves infusion drunk to stop diarrhea. Branches used as a toothbrush.	IR, 11, 15, 18, 70, 67, 73, 90
Sapindaceae, *Haplocoelum foliolosum* (Hiern) Bullock	Mariokwo (P)	SAJIT Z0048	S	Bushland	L	Rubbing	Leaf decoction used as an eye drop for infections.	15, 70, 73
Sapotaceae, *Pouteria adolfi-friedericii* (Engl.) A.Meeuse		SAJIT 006904	T		B	Boiling	Bark decoction drunk for stomachache.	IR, 15,

Scrophulariaceae, *Buddleja polystachya* Fresen.	Gelewa (M)	FOKP 11327	T	Bushland	R, L	Rubbing	Leaf and root juice applied into the eye for eye diseases.	IR, 18, 36
Solanaceae, *Solanum aculeastrum* Dunal	Sikawa (M)	FOKP 935	S	Cultivated	R	Boiling	Boiled decoction taken as a remedy for gonorrhea and for treating bronchitis.	IR, 18, 44, 71, 90
Solanaceae, *Solanum incanum* L.	Labotwa (M), Lopotwo (P), Maduranzura (L)	YMM/19/013	S	cultivated	R, L	Boiling	Root decoction used for fever, indigestion and stomach ache. Infusion of leaves applied to the ear as a remedy for earache. Roots chewed to reduce erectile dysfunction.	IR, 11, 15, 18, 30, 34, 40, 47, 61, 70, 67, 71, 68
Solanaceae, *Solanum nigrum* L.	Ksoya (P)	FOKP 11341	S	Cultivated	L, R	Boiling	Leaves boiled and eaten as vegetables. Chewing raw fruit treats stomach ulcers and general stomach problems.	61, 70, 73, 71
Solanaceae, *Solanum sessilistellatum* Bitter		FOKP 11379	S	Montane	R	Roasting	Roasted roots chewed and used as a cough remedy.	IR
Solanaceae, *Solanum terminale* Forssk.	Lobotwa (M)	SAJIT 004736	S	Riverine, Thickets, Forest margins	R	Boiling	Roots decoction drunk to cure colds and fever in adults.	34
Solanaceae, *Withania*	Tarkukai (M), Akakagh (P),	FOKP 14477	S	Escarpment, Lowland	R, L	Boiling	Root and leaf decoction used for skin rushes, relieves labor	15, 18, 34, 36, 42, 73, 71, 90

somnifera (L.) Dunal							pains, stomachache and amoebiasis.	
Stilbaceae, *Nuxia congesta* R.Br. ex Fresen.	Chorua (M)	SAJIT 005068	T	Highland, Bushland	B, L, R	Boiling, Chewing	Bark and leaves decoction used for indigestion, painful menstruation and as an astringent. Roots decoction administered for abdominal pains.	IR, 11, 18
Thymelaeaceae, *Gnidia latifolia* (Oliv.) Gilg	Nyalto (P),	SAJIT 006814	S	Woodland	B, R	Boiling	Root and bark concoction stimulate evacuation of bowels.	IR
Thymelaeaceae *Struthiola thomsonii* Oliv.		SAJIT 004793	S	Grassland	R	Boiling, Chewing	Chewing raw roots used as a cough remedy. Root infusion used as a remedy for stomach ache.	IR
Urticaceae, *Laportea alatipes* Hook.f.	Tobitiet (M)	FOKP 1738	H	Montane	L	Boiling	Leaf concoction drunk as a remedy for hepatic diseases.	15, 70
Urticaceae, *Laportea ovalifolia* (Schumach. & Thonn.) Chew		SAJIT 007125	H	Woodland	L, R	Pounding, boiling	Root concoction drunk to prevent excessive menstrual bleeding. Fresh leaves decoction drunk for easier removal of placenta after childbirth.	IR
Urticaceae, *Urtica massaica* Mildbr.	Kimelei (M)	FOKP 11312	H	Montane	R, L	Pounding, boiling	Roots boiled and decoction drunk for stomach ache. Leaves pound mixed with cold water as a remedy for ulcers.	IR, 15, 40, 44, 71

Verbenaceae, *Lantana trifolia* L.	Chemosong (M), Esimenenua (L)	FOKP 11322	S	Escarpment, Bushland	L, Br	Crushing, boiling	Pounded leaves used on ringworms. Leaves and branches decoction drunk for coughs.	IR, 11, 18, 30, 36, 47, 43, 61, 71
Verbenaceae, *Lippia javanica* (Burm.f.) Spreng.	Ngelepcha (M), Eru (P), Sulasula (L)	FOKP 11684	S	Highland, Bushland	L, Br, F	Sniffing, Boiling	Leaves and flowers sniffed to clear stuffy nose. Decoction of leaves and branches drunk for treatment of malaria and amoeba.	11, 18, 46, 71, 86, 90
Vitaceae, *Cissus rotundifolia* Vahl	Kwarkwarian (P), Ndabarwa (L)	SAJIT Z0041	C	Bushland	L, W	Pounding, Boiling	Leaves crushed and mixed with water and juice used to wash an infected ear. Whole plant decoction drunk for diarrhea and allergies.	67, 68
Vitaceae, *Cyphostemma adenocaule* (Steud. ex A.Rich.) Desc. ex Wild & R.B.Drumm.	Kantano (P), Lubombi (L),	YMM/19/162	C	Bushland	L, R	Heating, boiling	Roots boiled and drunk for treatment of syphilis and intestinal worms. Heated leaves placed on the chest of a person with pneumonia.	IR, 44, 61, 73, 86
Vitaceae, *Cyphostemma cyphopetalum* (Fresen.) Desc. ex Wild & R.B.Drumm.	Kiptora (M)	YMM/19/115	C	Bushland, Escarpment	L	Crushing	Leaves crushed and applied on enlarged glands.	IR, 11, 18

Vitaceae, *Cyphostemma serpens* (Hochst. ex A.Rich.) Desc.	Kirorot (M)	SAJIT 006876	C	Bushland, Grassland, Lowland	L, Br	Pounding	Leaves pounded and used for treatment of boils. Leaf and branches paste used for skin diseases.	11, 44
Vitaceae, *Rhoicissus tridentata* (L.f.) Wild & R.B.Drumm.	Taratwo (P), Lipwoni (L)	SAJIT 004846	S	Wooded grassland	R	Chewing	Roots concoction drunk to cure fever, abdominal and menstruation pains.	IR, 40, 44, 68
Viscaceae, *Viscum tuberculatum* A. Rich.	Torwa-cheptuywa (M)	YMM/19/084			B	Heating	Heated branches placed on the chest of a person with pneumonia.	IR, 44
Xanthorrhoeaceae, *Aloe volkensii* Engl.	Cherotwo (M), Tolkos (P), Linakha (L)	YMM/19/110	T	Escarpment, Bushland	L	Burning	Burnt leaves ash licked for whooping cough.	IR, 11, 15, 30, 44, 47, 68, 69
Xanthorrhoeaceae, *Aloe secundiflora* Engl.	Cherotwo (M), Tolkos (P), Eshikhaka (L)	YMM/19/083	S	Escarpment	L	Boiling	Leaves sap added to water and used for malaria, pneumonia, headache, and chest pains.	IR, 11, 15, 42, 51, 54
Zygophyllaceae *Balanites aegyptiaca* (L.) Delile	Tuyunwa (M), Tuyunwo (P),	YMM/19/098	T	Bushland, Grassland	R, L, S	Boiling, Pounding	Root and leaf concoction drunk for malaria, rheumatism and used as a blood cleanser. Seeds pound and boiled and drunk for respiratory disorders.	IR, 11, 15, 18, 61, 67, 90

Key: Parts used (PU): *L* Leaves, *R* Roots, *B* Bark, *Fl* Flower, *Fr* Fruit, *W* Whole plant, *Br* Branches, *Se* Seeds; Habit: *T* Tree, *S* Shrub, *H* Herb, *C* Climber, *L* Liana, *E* Epiphyte, *P* Parasite
